# The dose matters: Oral tofacitinib dose escalation or switching to deuruxolitinib for recalcitrant alopecia universalis and totalis, a retrospective case series

**DOI:** 10.1016/j.jdcr.2026.04.053

**Published:** 2026-05-02

**Authors:** Raymond Z. Ezzat, Meshi Paz, Caroline Kreytak, Maryanne M. Senna

**Affiliations:** a Department of Dermatology, Lahey Hospital and Medical Center, Burlington, Massachusetts; b Department of Dermatology, Harvard Medical School, Boston, Massachusetts

**Keywords:** alopecia areata, alopecia universalis, deuruxolitinib, deuruxolitinib 8 mg twice a day, JAK inhibitors, refractory, totalis, twice daily, tofacitinib, tofacitinib 10 mg twice a day

Alopecia universalis (AU) and alopecia totalis (AT), the most severe forms of alopecia areata, can be difficult to treat even with oral Janus kinase inhibitors (JAKi). Phase 3 trials show that baricitinib, ritlecitinib, and deuruxolitinib can achieve severity of alopecia tool (SALT) scores of 20 or less in many patients with severe alopecia areata.^1^, ^2^, ^3^ However, response rates are lower in long-standing AU or AT, particularly with longer disease duration.^4^ Clinical trials have also shown that increasing the dose of baricitinib from 2 mg to 4 mg may help convert some nonresponders, supporting a dose-response relationship.^4^ Prior open-label data also suggest a benefit from tofacitinib dose escalation from 5 mg twice daily to 10 mg twice daily in some nonresponders with severe alopecia areata (AA).^5^ To our knowledge, few reports have described outcomes in long-standing AU or AT after either escalation to tofacitinib 10 mg twice daily or switching to deuruxolitinib 8 mg twice daily after failure of prior JAKi therapy.

We conducted an IRB-approved retrospective case series at a tertiary hair disorders clinic. Eligible adults with AA who had a SALT score of at least 95 at our center, had received at least 1 prior systemic therapy, and had a minimum of 6 months of follow-up after JAKi regimen modification were included. The regimen modifications of interest were escalation to tofacitinib 10 mg twice daily or transition to deuruxolitinib 8 mg twice daily after inadequate response to prior systemic JAKi therapy.

Three patients met the inclusion criteria (Table I). All experienced AU with complete scalp and body hair loss at key times and had disease durations of 6 to 9 years. Case 1 had previously received oral baricitinib 4 mg daily for 12 months without sufficient hair regrowth (SALT 100). Case 3 had been treated with baricitinib 4 mg daily for 16 months, followed by ritlecitinib 50 mg daily for 7 months, but still had extensive scalp AA involvement. Case 2 has been on tofacitinib 5 mg twice daily for 9 months, then on oral tofacitinib 15 mg once daily for an additional 10 months, with insufficient hair regrowth (SALT 100). All 3 patients also received concomitant low-dose oral minoxidil, which may have contributed to regrowth, although each had persistent severe disease before the JAKi regimen change.

**Table I:** Baseline characteristics and prior therapies in 3 adults with refractory alopecia universalis treated with twice-daily oral JAK inhibitors

Variable	Case 1	Case 2	Case 3
Age	33	18	20
Sex	Female	Female	Male
Phenotype	AU	AU	AU
Duration of current severe episode	∼7 y	∼6–7 y	∼9 y
Baseline SALT at specialty clinic	100	100	100
Autoimmune comorbidities	Vitiligo, eczema, and allergic disease	None	Ulcerative colitis and erythema nodosum
Family autoimmune history	Hypothyroidism, UC, and AU in relatives	None reported	AA in cousin and RA in mother
Key prior systemic therapies	Cyclosporine and prednisone	Systemic steroids	Prednisone and hydroxychloroquine
Prior JAK exposure	1. Baricitinib 4 mg/day for 12 mo, no regrowth (SALT 100)	1. Tofacitinib 5 mg twice a day for 9 mo 2. Tofacitinib 15 mg every day for 10 mo. SALT remained at 100 throughout both regimens.	1. Baricitinib 4 mg/day for 16 mo, partial response at SALT 50. 2. Ritlecitinib 50 mg/day for 7 mo with an inadequate response. SALT remained at 50.

*AA,* Alopecia areata; *AU,* Alopecia universalis; *JAK*, Janus kinase inhibitors; *RA*, rheumatoid arthritis; *SALT*, severity of alopecia tool; *UC*, ulcerative colitis.

After initiation of tofacitinib 10 mg twice daily in Case 1, SALT scores improved from 100 to 0 within ∼6 months and remained at 0 at 12 months. In Case 2, SALT decreased from 100 to 15 within 12 months. In both cases, improvement followed escalation to tofacitinib 10 mg twice daily.

Finally, in Case 3, after transitioning to deuruxolitinib 8 mg twice daily, SALT decreased from 50 to 20 over ∼10 weeks of follow-up (Table II). This relatively rapid improvement after sequential failure of baricitinib and ritlecitinib is clinically notable and may suggest benefit from switching within class in selected refractory patients.

**Table II:** JAKi regimens, SALT trajectories, and safety

Variable	Case 1	Case 2	Case 3
High intensity regimen	Tofacitinib 10 mg twice a day	Tofacitinib 10 mg twice a day	Deuruxolitinib 8 mg twice a day
Concomitant LDOM	1.25 → 2.5 mg every day	1.25 → 2.5 mg every day	5 mg every day
SALT at start of high intensity	100	100	50
SALT at ∼6 mo	0	85	30
SALT at ∼12 mo	0	15	20
Serious infections	None	None	None
MACE/VTE/malignancy	None	None	None
Treatment-limiting AEs	None	None	None

*AE*, Adverse events; *JAKi*, Janus kinase inhibitors; *LDOM*, low-dose oral minoxidil; *MACE*, major adverse cardiovascular events; *SALT*, severity of alopecia tool; *VTE*, venous thromboembolism.

No major adverse cardiovascular events, venous thromboembolism, malignancies, or serious infections occurred during the follow-up period. No treatment-limiting laboratory abnormalities or dose reductions due to toxicity were reported. Given the boxed warnings associated with oral JAK inhibitors, especially for major adverse cardiovascular events, venous thromboembolism, serious infections, and malignancies, these short-term findings should be interpreted with caution.

This small case series suggests that in recalcitrant, severe AA, escalation to tofacitinib 10 mg twice daily or switching to deuruxolitinib 8 mg twice daily after previous JAK inhibitor failure can result in clinically meaningful hair regrowth. These results align with 2 related clinical strategies: dose escalation and switching within the same class. Limitations include concomitant oral minoxidil use; retrospective, single-center design; small sample size; lack of a control group; and short follow-up. More comprehensive data are needed to determine which refractory phenotypes benefit most from dose escalation or within-class switching and to evaluate the additional risks compared to on-label dosing.

## Conflicts of interest

None disclosed.
